# Pore-scale mechanisms of CO_2_ storage in oilfields

**DOI:** 10.1038/s41598-020-65416-z

**Published:** 2020-05-22

**Authors:** Abdulla Alhosani, Alessio Scanziani, Qingyang Lin, Ali Q. Raeini, Branko Bijeljic, Martin J. Blunt

**Affiliations:** Imperial College London, Department of Earth Science and Engineering, SW7 2AZ London, UK

**Keywords:** Climate-change mitigation, Carbon capture and storage

## Abstract

Rapid implementation of global scale carbon capture and storage is required to limit temperature rises to 1.5 °C this century. Depleted oilfields provide an immediate option for storage, since injection infrastructure is in place and there is an economic benefit from enhanced oil recovery. To design secure storage, we need to understand how the fluids are configured in the microscopic pore spaces of the reservoir rock. We use high-resolution X-ray imaging to study the flow of oil, water and CO_2_ in an oil-wet rock at subsurface conditions of high temperature and pressure. We show that contrary to conventional understanding, CO_2_ does not reside in the largest pores, which would facilitate its escape, but instead occupies smaller pores or is present in layers in the corners of the pore space. The CO_2_ flow is restricted by a factor of ten, compared to if it occupied the larger pores. This shows that CO_2_ injection in oilfields provides secure storage with limited recycling of gas; the injection of large amounts of water to capillary trap the CO_2_ is unnecessary.

## Introduction

**Figure 1:**
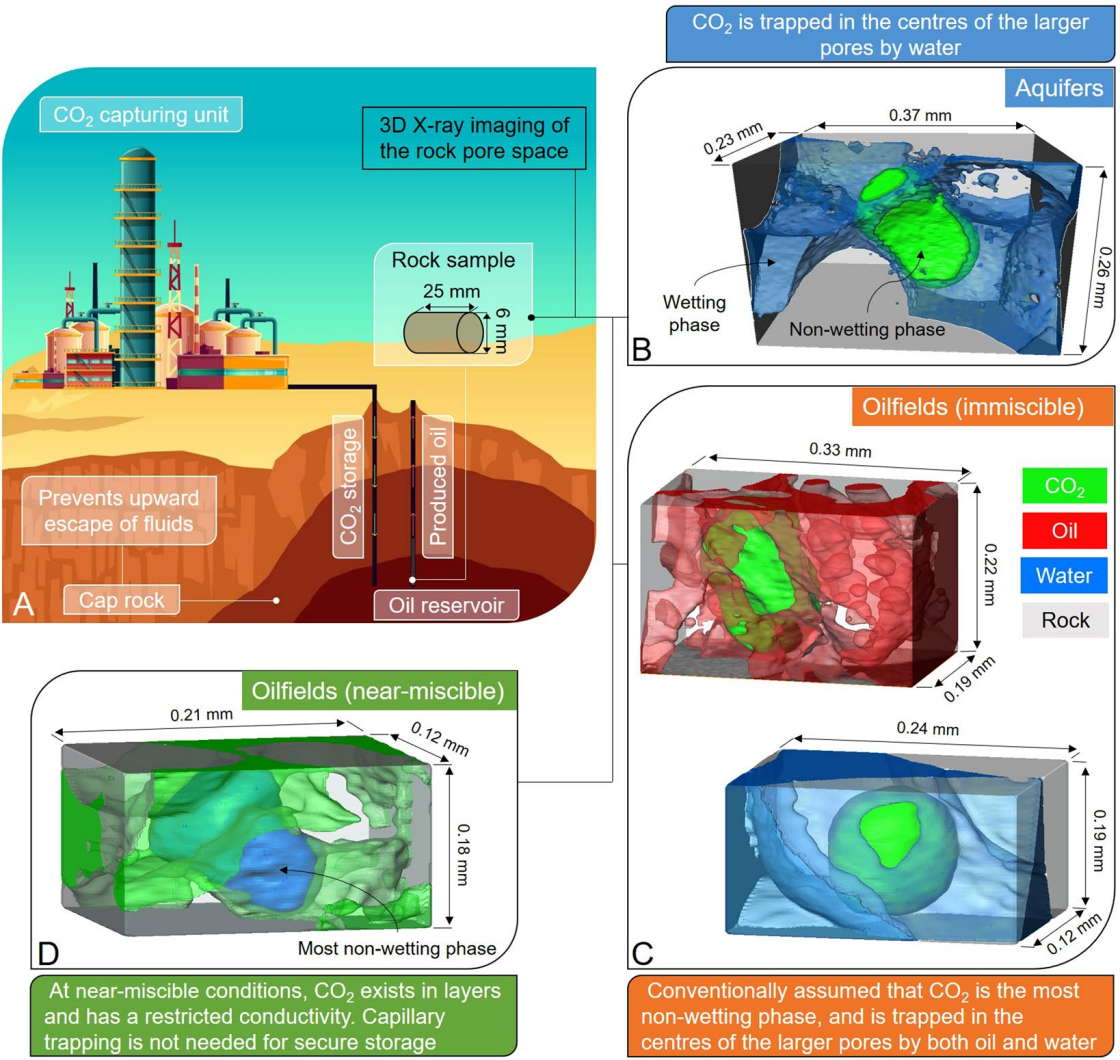
Diagram showing carbon capture and storage coupled with enhanced oil recovery. (**A**) A schematic of CO_2_ storage in depleted oil reservoirs. The behaviour of CO_2_ underground is controlled by pore-scale processes. (**B**–**D**) show real images of reservoir fluids trapped in a single pore of carbonate rocks at elevated temperatures and pressures. CO_2_ is shown in green, water in blue, oil in red, while the rock is rendered transparent. (**B**) In a saline aquifer, CO_2_ is the non-wetting phase and occupies the larger pores. To restrict its flow, it must be capillary trapped through displacement with water^19^. (**C**) In an oilfield under immiscible conditions, gas (CO_2_) is also the most non-wetting phase: to restrict its flow it must be capillary trapped by either oil (top) or water (bottom). (**D**) However, we normally encounter near-miscible conditions, where CO_2_ exists in layers surrounding the water phase, which is now most non-wetting. CO_2_ flow is restricted in these layers. Here, only CO_2_ and water are shown for clarity. The 3D pore-scale images were acquired by X-ray micro-tomography with a voxel size of approximately 2 µm, and visualized using Avizo 9.5 software (https://www.fei.com/software/amira-avizo/).

With anthropogenic CO_2_ emissions into the atmosphere of 37 GtCO_2_ in 2018^1^, fuelled by the growth in fossil fuel consumption^2^, any viable solution to avoid dangerous climate change has to involve the rapid and large-scale implementation of CO_2_ capture and storage (CCS)^3–9^. Although there are abundant CO_2_ storage sites in deep saline aquifers^10^, given the short time frame to implement the technology at a global scale, geological storage of CO_2_ in the next decade is most practical in depleted oil and gas reservoirs, where the infrastructure including facilities, pipelines, and injection wells, as well as detailed knowledge of the fields already exists, combined with an immediate financial incentive from enhanced oil recovery (EOR)^11–14^, see Fig. 1.

In CO_2_-EOR projects, the injection of CO_2_ into depleted oil and gas reservoirs can result in an additional hydrocarbon recovery, which may offset some of the cost of CO_2_ capture and storage^15–17^. While depleted oil and gas reservoirs are well known for their commercial quality permitting investment into large-scale EOR-CCS projects, they typically contain abandoned boreholes, which can potentially be conduits for rapid escape of stored CO_2_.

How should CO_2_ injection be designed to maximize storage security? While CO_2_ underground migrates over several kilometres, the physical processes that control its movement occur at the micron-scale of the pores within the rock^18^. In saline aquifers, subsequent to the injection of CO_2_, water imbibes back into the pore space either at the trailing edge of a rising CO_2_ plume or through engineered water injection. Since water wets the rock surfaces in saline aquifers, water flows through wetting layers, leaving CO_2_, the non-wetting phase, stranded in the centres of the larger pores in disconnected blobs, see Fig. 1B; hence a significant amount of CO_2_ can become trapped in the subsurface^19,20^. This capillary, or residual, trapping is important, as otherwise CO_2_ remains connected through these wider pores, facilitating flow and potential escape. Capillary trapping limits the movement of CO_2_ and is the most rapid and effective mechanism to ensure safe storage for regionally extensive saline aquifers^21^.

In oilfields, CO_2_ is often injected at conditions designed to be nearly, but not completely, miscible with the oil, to improve oil recovery^22–26^. In this case, it is conventionally assumed that CO_2_ is the most non-wetting phase and flows rapidly, preferentially filling the larger pore spaces, while oil and water occupy the smaller pores^18,27,28^. Similar to aquifers, it is believed that CO_2_ movement can be prevented through capillary trapping by both oil and water regardless of the rock wettability^28–30^, see Fig. 1C. To maximize both oil recovery and CO_2_ storage, it is considered that CO_2_ and water should be injected together to restrict the flow of CO_2_ to oil production wells^31,32^.

To better characterize the mechanism of CO_2_ storage in oilfields, we use X-ray imaging to visualize the pore structure and fluids at micron resolution inside the rock in three dimensions^33^. We investigate the pore-scale distribution of CO_2_, oil and water at conditions representative of oilfields. The pore-scale configuration of fluids determines the flow of CO_2_.

In this work, we challenge the assumption that CO_2_ remains the most non-wetting phase in oilfields, where crude oil has rendered rock surfaces oil-wet^34,35^. Oil and CO_2_, at the high pressures and temperatures encountered underground, are typically near miscible with a low interfacial tension of approximately 1 mN/m^27^: oil and CO_2_ have similar wetting properties and pore-scale configuration^36^. The wettability order is different such that CO_2_ is no longer the most non-wetting phase, instead it spreads in layers as the intermediate-wet phase, see Fig. 1D, with significant implications for trapping, flow and storage. The existence of CO_2_ in layers substantially impedes its movement in the reservoir, and its possible escape through boreholes, which eliminates the need for water injection to restrict its flow, and hence more of the pore space can be occupied by CO_2_, boosting storage capacity.

In this paper we propose a different pore-scale storage mechanism to that in saline aquifers. In aquifers, where the CO_2_ is non-wetting, it can be safely trapped as bubbles or ganglia in the larger pores. In oilfields, however, this trapping does not occur, as the CO_2_ is not the most non-wetting phase. Instead, we have a different way to restrict CO_2_ movement, by confining the CO_2_ to layers in the pore space, which means that the CO_2_ can only flow very slowly.

## Results and Discussion

We used X-ray micro-tomography (micro-CT) to image CO_2_, oil and water in the pore space of a reservoir rock under oil-wet near-miscible conditions at high temperature, 70 °C, and pressure, 10.85 MPa. The rock sample was extracted from a giant producing carbonate oil reservoir in the middle east and restored to the initial reservoir wetting conditions by dynamically and statically saturating the sample using crude oil in a process called ageing. More details are provided in the Supplementary Material. Similar samples have been used previously to study water flood recovery^37,38^. We selected a water-alternating-gas (WAG) flooding scheme to mimic the typical displacement sequence encountered in oil reservoirs. The injection sequence into the oil saturated rock was: (i) first water flooding [WF1]; (ii) gas injection [GI], and (iii) second water flooding [WF2]. We will demonstrate where and how readily CO_2_ flows in the rock pore space by: (i) quantifying the configuration of the three phases; (ii) calculating the flow conductance of the phases; and (iii) measuring the amount of CO_2_ trapping.

### Distribution of CO_2_, oil and water in the pore space

**Figure 2:**
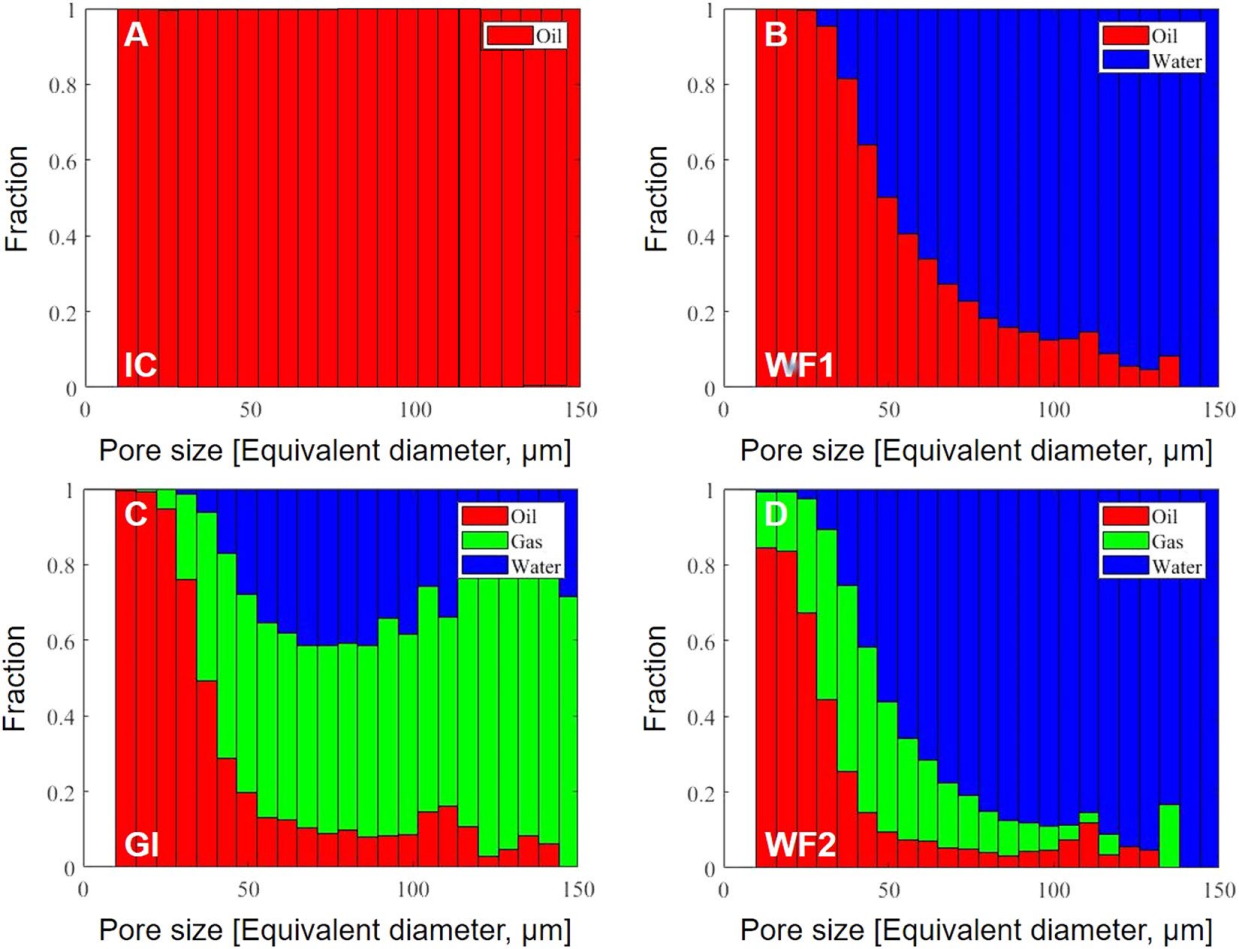
Bar charts showing the fraction of the pores occupied by each phase as a function of pore diameter after each injection step. Pore occupancy is defined as the phase in the centre of the pore – this phase will also have the largest volume in the pore. (**A**) Initial reservoir conditions (there is an initial water saturation of ~1% which is not evident from the bar chart) [IC], (**B**) first water flooding [WF1], (**C**) gas injection [GI], and (**D**) second water flooding [WF2]. Oil is shown in red, water in blue and gas, CO_2_, in green.

To understand flow and displacement we first quantify pore occupancy, see Fig. 2. The size of pores each fluid occupies controls how readily it flows in the pore space: if a fluid fills larger pores it flows more readily as opposed to filling smaller pores. We performed a topological analysis of the pore space to identify the widest regions (pores) that are connected by narrower restrictions: the pore size is the diameter of the largest sphere that fits in a pore; the centre of the sphere is the centre of the pore. The phase occupancy is defined as the phase which resides at the centre of the pore^29,36,39^. We find that water is the most non-wetting phase since it occupies the largest pores, followed by gas, with oil most wetting in the smallest pores. This wettability order from most to least wetting – oil, gas, water – has been seen in micro-model experiments^40^ but not inside reservoir rocks before, and contradicts previous studies where the oil and gas properties were not representative of subsurface CO_2_ storage conditions^28,29^.

Initially, the rock was saturated with oil to restore the initial reservoir conditions, see Fig. 2A: at initial conditions (IC) some water was present in the pore space but at a very low saturation ~ 1%, which is why it is not evident on the bar chart. As expected for oil-wet media, during the first water flooding (WF1), water, the non-wetting phase, invaded the largest pores, while oil, the wetting phase, remained confined in the smaller ones. After gas injection (GI), water was trapped in the centres of the pore space as disconnected ganglia, Fig. 1D. In water-occupied pores, the gas can reside in layers next to the solid trapping water in the centre, see Fig. 5. The wettability order – oil, gas, water – is more apparent during the second water flooding (WF2), where water invaded the centres of the larger pores confining gas and oil to intermediate and small sized pores respectively. This shows that the injection of water in WF2 limits the available storage capacity for CO_2_ as the usable volume of the oilfield pore space occupied by CO_2_ is restricted.

### CO_2_ flow conductivity

**Figure 3:**
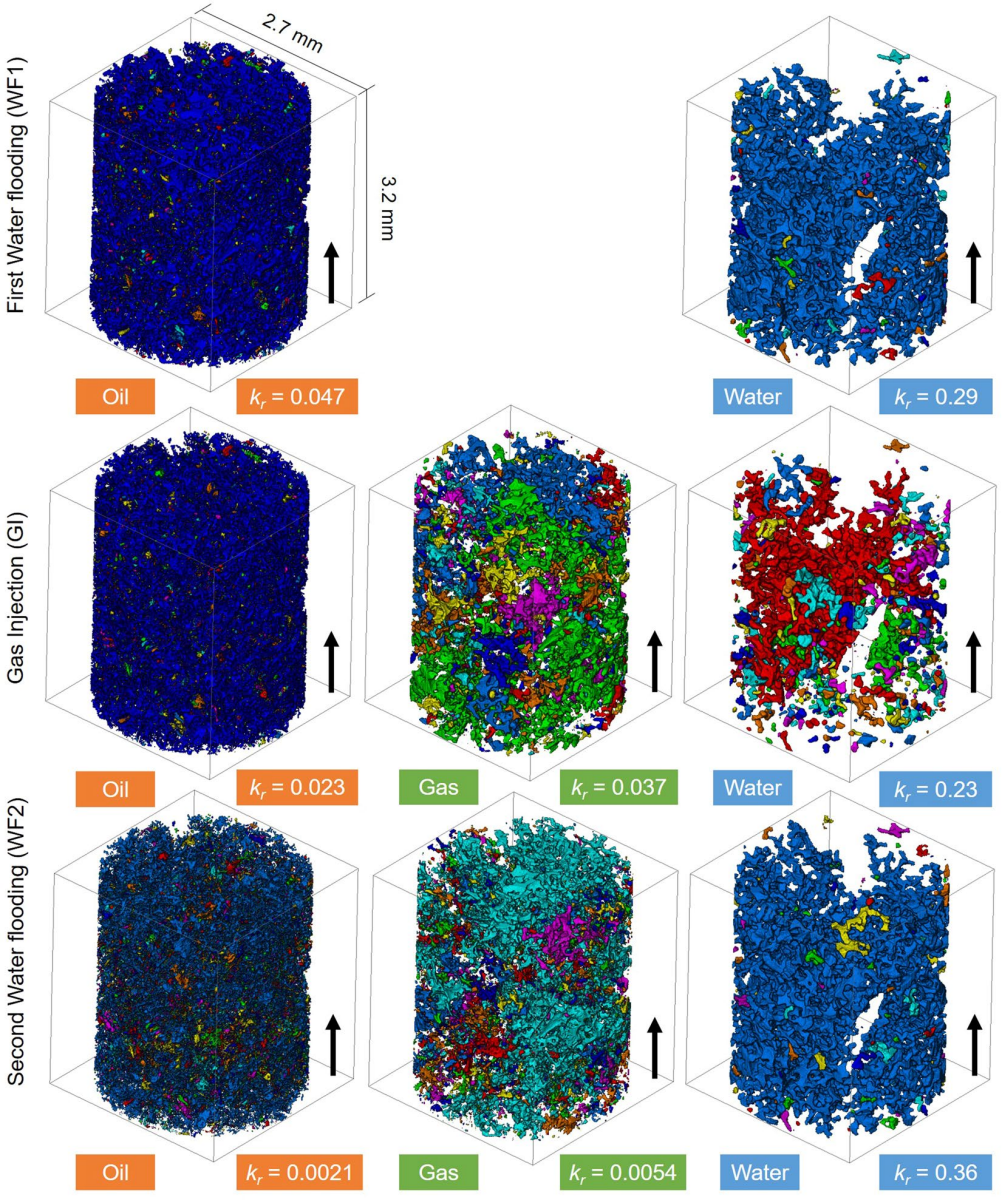
Three-dimensional maps showing the connectivity of the phases during the displacement sequence. The colours indicate discrete clusters of each phase. The relative permeability, *k*_*r*_, is shown in the boxes. The oil, gas and water phase distributions were obtained by imaging at a high-resolution, 1.82 µm/voxel, the rock pore space during the experiment, while the relative permeability values were computed from flow field simulations. The connectivity maps were plotted using Avizo 9.5 software (https://www.fei.com/software/amira-avizo/).

Figure 3 shows the connectivity of the phases after each injection step. During water injection, water can form a connected path through the larger pores. In contrast, oil and gas tend to form thinner structures. Oil fills the smaller pores and is retained close to the solid surface, while CO_2_ forms layers which follow a tortuous path through the pore space.

**Figure 4:**
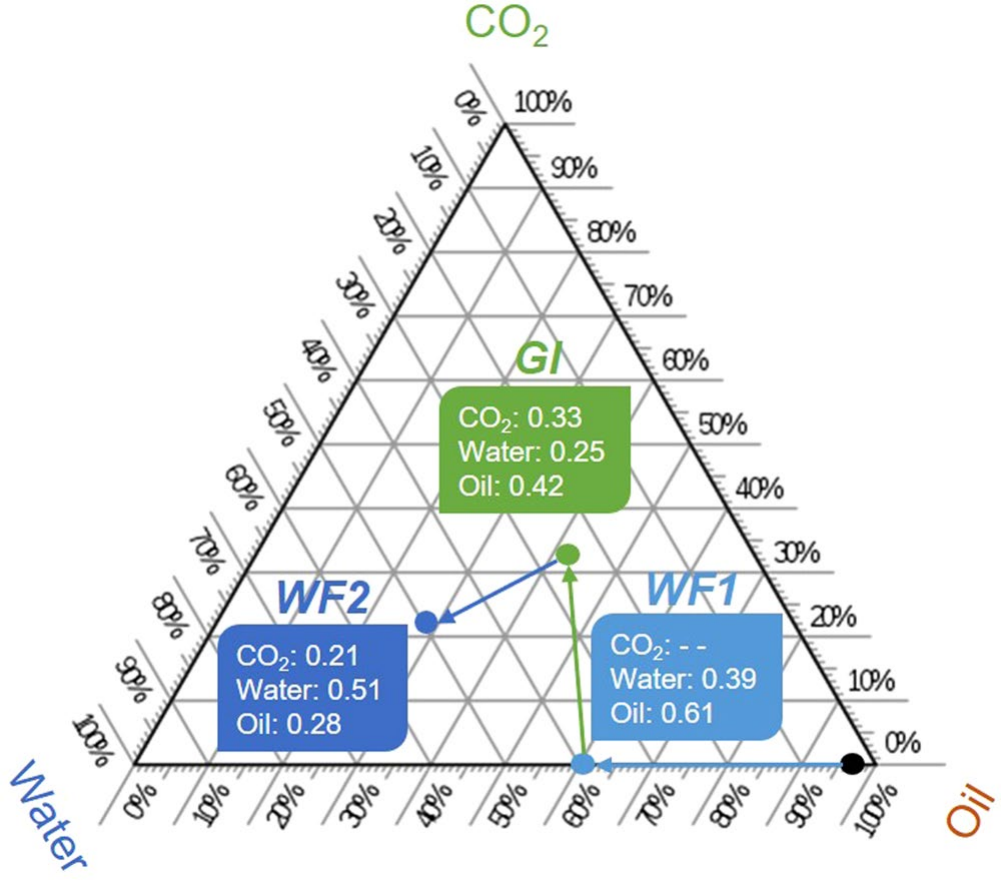
A three-phase ternary diagram showing the end-point saturations during the flooding sequence. Saturation is defined as the volume of a phase divided by the total pore volume. At first (black point on the diagram), the rock is restored to its initial reservoir conditions (water saturation: 0.01 and oil saturation: 0.99). The coloured arrows point to the chronological order of injection events: (i) first water flooding [WF1]; (ii) gas injection [GI], and (iii) second water flooding [WF2]. The error in the saturation measurement is within ± 2%.

We computed the flow field, that is the velocity, of oil, gas and water on the images shown in Fig. 3. The simulations were performed on the largest connected phase cluster in each of the 3D images with 1483 × 1483 × 1000 voxels, see Supplementary Fig. S4. From these flow fields, the permeability of each phase can be computed. When the pore space is completely filled with one phase the absolute permeability is 3.7 × 10^−13^ m^2^; this value is close to the directly-measured value of 2.7 × 10^−13^ m^2^ through the whole sample. The relative permeability is defined as the ratio of the permeability of a phase that partially fills the pore space to the absolute permeability^18^. As indicated in Fig. 3, the relative permeability of gas is only 0.037 after gas injection despite having a saturation of 33 ± 2%, see Fig. 4 – the flow conductance is reduced by a factor of almost 30, while water in the larger pores has a higher relative permeability of 0.23. The oil has the lowest relative permeability of only 0.023 since it occupies the smallest pores. This reduction in oil and gas relative permeability under near-miscible oil-wet conditions has been observed in conventional flow tests on rock samples, but not explained using the wettability order^23^. After WF2, the gas and oil relative permeabilities are further reduced by one order of magnitude to 0.0054 and 0.0021 respectively, implying extremely restricted flow; however, less CO_2_ is stored in the pore space with a saturation of only 21 ± 2%, see Fig. 4.

### CO_2_ saturation and trapping

The remaining saturation of CO_2_, oil and water in the rock pore space after each injection was measured on segmented images of the whole rock. The change in fluid saturations throughout the flooding sequence is shown on the ternary diagram in Fig. 4. After the first water flooding (WF1), only 39 ± 2% of the oil was recovered. This is ascribed to water invading the centres of the pores, while oil remains connected in the corners and small pores in wetting layers, Fig. 3. This is a common characteristic of oil-wet media^38,41,42^. During gas injection (GI), CO_2_, the intermediate-wet phase, displaced some of the oil that was in the corners of the pore space leaving water stranded in the centres. This is ideal from an oil recovery standpoint: the recovery factor increased to 58 ± 2%. Almost 33 ± 2% of the pore space remained saturated by CO_2_ after gas injection (GI). WF2 displaced both oil and CO_2_. Despite a substantial increase in the oil recovery during WF2 (14 ± 2%), as seen in other WAG experiments^28,40^, it results in a lower CO_2_ saturation (21 ± 2%), i.e. a lower CO_2_ storage capacity after water injection: less CO_2_ is stored and indeed some of the injected CO_2_ is produced with the oil. This is also a lower CO_2_ saturation than is capillary trapped under water-wet conditions in similar rocks^19^.

### Gas layer thickness

**Figure 5:**
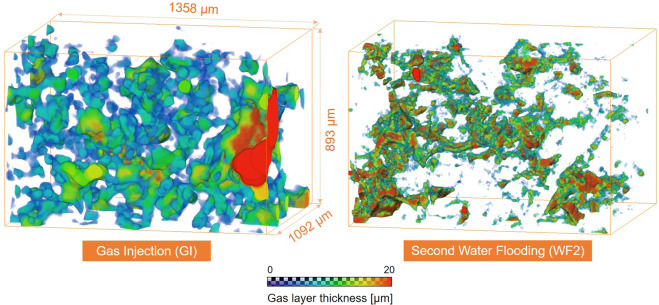
Three-dimensional maps of the local thickness of gas layers computed after (left) gas injection (GI) and (right) second water flooding (WF2). The thickness maps were quantified on images of voxel size of 1.82 µm with 746 × 491 × 600 voxels. The gas layers have an average thickness of approximately 15–20 µm. The layers were visualized using Avizo 9.5 software (https://www.fei.com/software/amira-avizo/).

Figure 5 shows maps of the local thickness of the gas layers after gas injection (GI) and second water flooding (WF2). The average saturation was approximately constant across the sample after each injection. The thickness of these gas layers was quantified on images with 746 × 491 × 600 voxels of size 1.82 µm: the thickness is defined as the diameter of the largest sphere that fits entirely in the gas phase at each location. Note that the thickness in both cases is much smaller than a typical pore size, see Fig. 2 where the x-axis of the bar chart shows the pore size distribution of the rock sample used, indicating that the gas resides principally in layers, as opposed to occupying the centres of larger pores.

It is common practice to inject alternate slugs of water and gas in oilfield operations for three reasons. Firstly, this saves money, as the gas is more expensive to collect and inject than water. Secondly, it is presumed that gas is the most non-wetting phase: injecting water helps to limit gas flow and production^27^. Thirdly, repeated flooding cycles lead to higher oil recovery, as we see here, Fig. 4. We have shown that for storage applications, CO_2_ flow is restricted even when gas alone is injected. To maximize storage capacity, the CO_2_ saturation should be as high as possible. This implies that an injection strategy of CO_2_ injection only, or with limited amounts of water, is favourable, and that CO_2_ is unlikely to be produced in significant quantities. Moreover, we have shown that CO_2_ has very low relative permeability in the reservoir, which makes it harder – but not impossible – for the stored CO_2_ to flow towards abandoned wells and escape through legacy boreholes. Therefore, after CO_2_ injection, the wells must be closed in and the CO_2_ should remain underground for thousands of years, since its upwards migration is prevented by caprock^6^, see Fig. 1.

## Conclusions

To help meet the global requirement to reduce CO_2_ emissions, we have investigated CO_2_ storage in oilfields. We have demonstrated that it is wettability order that controls the flow of fluids in the reservoir. Our results prove that the traditional assumption that CO_2_ is always the most non-wetting phase is incorrect. The injection of CO_2_ results in near-miscible conditions, where oil is the most wetting phase, occupying the smallest pores as well as wetting layers, water is the most non-wetting phase, residing in the centres of the pores, while CO_2_ is the intermediate-wet phase, forming layers in the corners of the pore space. This wettability order leads to the confinement of CO_2_ in layers of low flow conductivity. Therefore, the injection of water is not required to constrain the gas flow. The migration of CO_2_ beyond the oilfield is prevented by the cap rock, which has contained the hydrocarbons for millions of years.

We suggest that near-miscible conditions in reservoir rocks are ideal for recovery and storage: the wettability order oil-gas-water is likely to be seen in any oil-wet or mixed-wet rock, which comprise the majority of carbonate reservoirs^18^. On the other hand, if complete miscibility is achieved, the oil and gas flow together as one phase^43^. While this is favourable for oil recovery, it facilitates the flow of CO_2_ and so is less favoured for secure storage. Our experiment has considered only one rock type and mineralogy – a heterogenous reservoir calcite – but we suggest that the same trends in local displacement efficiency will be observed in any system with this wettability order and near-miscible conditions such that gas can form spreading layers. Future work could study a wider range of rock types, including sandstones, where the wettability may be different, even for the same fluids.

An engineering assessment of CO_2_ storage in oilfields will also involve a quantitative analysis of large-scale flow accounting for reservoir structure, variations in permeability and well placement. However, one key input is the flow potential of the phases, controlled by pore-scale fluid configurations: we have shown that the flow of CO_2_ is impeded by an order of magnitude or more compared to conventional models where it is the non-wetting phase. This finding implies that CO_2_ can be injected alone, or with minimal amounts of water, to maximize storage capacity while still restricting the possibility of CO_2_ escape.

## Methods

### Reservoir rock, wettability restoration and fluid properties

The rock selected was a carbonate extracted from a giant producing oil reservoir in the middle east (composition: 96.5 wt% calcite): the sample was a cylinder of diameter 5.9 mm and length 24.9 mm. The rock was restored to the initial reservoir wetting conditions by a process called ageing. The rock, initially brine-saturated, was flooded continually for four weeks with crude oil (an oil from the same reservoir as the rock, surface density = 830 kg/m^3^) at high temperature, 80 °C, and pressure, 10 MPa. We have used reservoir brine to represent the water phase, decane to represent the oil phase and CO_2_ in a supercritical state to represent the gas phase in the experiment. Sodium iodide (NaI) and iododecane (C_10_H_21_I) solutions were added to the water and oil phases with concentrations of 30% and 20% respectively to obtain effective phase contrast in the micro-CT images. For a more detailed description of the rock and fluid properties and the wettability restoration process please refer to the Supplementary Information.

### X-ray micro-tomography imaging and flow experiment

A ZEISS Xradia 510 Versa micro-CT scanner was used to acquire high-resolution three-dimensional images of the reservoir rock and the fluids within it. The rock was inserted in a Hassler type carbon fibre coreholder that is X-ray transparent and placed inside the micro-CT for image acquisition. The coreholder was connected to flow lines to perform the injection sequence: (i) first water flooding [WF1]; (ii) gas injection [GI]; and (iii) second water flooding [WF2]. Prior to starting the injection sequence, the crude oil in the restored sample was completely displaced and replaced by doped oil at the experimental conditions, 70 °C, and 10.85 MPa. During each injection, 1 pore volume, 0.708 mL, of fluid was injected into the sample at rate of 0.005 mL/min. Images of the whole rock sample (1652 × 1652 × 66974 voxels) were acquired after each injection with a voxel size of 3.57 µm to characterize the pore occupancy and measure fluid saturations. A scan of higher resolution (1483 × 1483 × 1758 voxels), with a voxel size of 1.82 µm, was also acquired at the centre of the rock to examine gas layers. The images were acquired 2 h after each injection to allow the fluids in the rock pore space to reach equilibrium conditions. After the experiment, the rock was cleaned to remove any organic material by injecting toluene into the pore space, followed by the injection of deionized water, and then dried at 60 °C in vacuum for three days to prepare the sample for acquisition of a dry scan. This was done after the experiment for convenience so that all the images could be acquired during the same period. A more detailed description of the flow apparatus, experimental procedure and image acquisition can be found in the Supplementary Information.

### Image processing and segmentation

The raw micro-CT images acquired were processed for phase identification, i.e. image segmentation. The images were segmented into four phases: water, rock, oil and gas. The 3.57 µm voxel images of the whole sample were segmented using the seeded watershed algorithm^44^. A non-local means filter was applied to the 3.57 µm voxel images to smooth the grey scale images and improve the watershed segmentation^45^. The higher resolution images, 1.82 µm voxel size, were segmented using machine learning-based trainable WEKA segmentation method^46^. WEKA segmentation was chosen to segment the high-resolution images as it preserves the shape of the interface between the phases, facilitating more accurate characterization of flow properties and thicknesses of gas layers^38^. However, WEKA is very CPU intensive, hence, it was not possible to apply it to the large 3.57 µm voxel size images of the whole sample. A more detailed description of the image segmentation is provided in the Supplementary Information.

### Flow simulation

We computed flow on segmented high-resolution images of size 2.2 × 10^9^ voxels. We treated each phase separately and solved the Navier-Stokes equation using a finite volume method^47^ with a constant pressure differential across the image in the experimental direction of flow, and with no flow boundaries with the solid and the interfaces with other phases. The no-flow boundary condition removes complexities in the calculation, so that the flow of one phase is not dependent on the flow rate and direction of the other phases. This is also a good approximation for layer flow of CO_2_ since it is much less viscous than the oil or water (see, for instance, Shams, *et al*.^48^ for a quantification of these effects in simple geometries). The permeability of the phase was computed from the ratio of flow rate to pressure gradient using Darcy’s law. For more details refer to the Supplementary Information.

## Supplementary information

Supplementary information.
