# Supplementary material for: Pore-scale mechanisms of CO2 storage in oilfields
Source: Sci Rep. 2020 May 22;10:8534. doi: 10.1038/s41598-020-65416-z (PMC7244489; doi:10.1038/s41598-020-65416-z)
Supplement: Supplementary file 1 — Supplementary information. [file 41598_2020_65416_MOESM1_ESM.docx]

#### Supplementary Information

Pore-scale mechanisms of CO_2_ storage in oilfields

Abdulla Alhosani^a^*, Alessio Scanziani^a^, Qingyang Lin^a^, Ali Q. Raeini^a^, Branko Bijeljic^a^, Martin J. Blunt^a^

*^a^Imperial College London, Department of Earth Science and Engineering, SW7 2AZ, London, UK*

* *Correspondence*: [Abdulla.alhosani17@imperial.ac.uk](mailto:Abdulla.alhosani17@imperial.ac.uk)

**Rock and fluid properties**

The rock sample used in this study was extracted from a large producing carbonate oil reservoir in the middle east which is mainly calcite (96.5% wt), with small amounts of dolomite, kaolinite and quartz[^1^](#_ENREF_1). The sample was 5.9 mm in diameter and 24.9 mm in length with a total helium porosity of 0.26 and a segmented porosity, from the X-ray images, of 0.25. Sample pore volume was measured to be 0.708 mL based on the helium porosity. The absolute permeability was measured to be 2.7×10^-13^ m^2^ on a larger rock (3.8 cm in diameter and 6.4 cm in length) from which our sample was drilled.

The three fluid phases selected to perform the experiment were: (i) supercritical (sc) CO_2_ as the gas phase, (ii) decane as the oil phase, and (iii) brine (water containing the same salt composition as water from the same reservoir as the rock) as the water phase. A 30% wt sodium iodide solution and a 20% wt iododecane solution were added to the water and oil phases respectively, to enhance the contrast between the fluid phases in the micro-CT images in a process called doping. Doping allows us to distinguish between the oil, water and gas phases in the raw images and is essential to obtain an accurate segmentation of the fluids and rock. At the selected experimental conditions, 70 ^o^C and 10.85 MPa, the oil and gas phases have an interfacial tension of 1 mN/m, measured using the pendant drop method[^2^](#_ENREF_2), resulting in near-miscible conditions between the fluids. Fluid properties are listed in Table S1.

Table S1

Thermophysical properties of the three fluid phases selected for the experiment at 70^o^C and 10.85 MPa. Data from [Georgiadis, et al. ^3^](#_ENREF_3), [Heidaryan, et al. ^4^](#_ENREF_4), and [NIST ^5^](#_ENREF_5). *Densities measured at ambient conditions, 20^o^C and 0.1 MPa. The interfacial tension between oil and gas was measured directly using the pendant drop method[^2^](#_ENREF_2).

| Fluid | Composition (%wt) | $\rho$ (kg$\cdot$m^-3^) | $\mu$ (mPa$\cdot$s) | $\sigma$ (mN$\cdot$ m^-1^) |
| --- | --- | --- | --- | --- |
| Water | 70% brine + 30% Sodium Iodide | 1414.9* | 0.468 | $\sigma_{gw}$= 31 |
| Oil | 80% decane + 20% Iododecane | 796.8* | 1.12 | $\sigma_{ow}$= 30 |
| Gas | scCO_2_ | 247.7 | 0.023 | $\sigma_{go}$= 1 |

**Reservoir initial wettability restoration (ageing protocol)**

The rock sample was restored to the initial reservoir conditions prior to performing the experiment through a process known as ageing. Ageing is performed by exposing the rock surfaces to crude oil either dynamically and/or statically. Dynamic ageing refers to the continuous injection of the crude oil inside the rock sample, while static ageing means no flow (the sample is typically immersed in a crude oil bath): we used a combination of dynamic and static ageing. We have used a light crude oil from the same reservoir as the rock in the ageing process, see Table S2 for the crude oil properties[^1^](#_ENREF_1).

Prior to ageing the rock, it was cleaned using methanol and dried in an oven for 24 h. Reservoir conditions, 80 ^o^C and 10 MPa, were then established and the dry sample was saturated with formation brine (100% brine saturation): this formation brine was pre-equilibrated with carbonates to ensure that no chemical reactions occurred in the rock. The first step involved the injection of crude oil into the sample, where 40 pore volumes (1 pore volume = 0.708 mL) of crude oil were injected from the bottom of the sample, with a step wise increase in the flow rate from 0.001 mL/min to 0.1 mL/min. The same procedure was then repeated; however, crude oil was injected from the top of the sample this time. Once the sample was saturated with crude oil, the dynamic ageing procedure started, where five pore volumes of crude oil were injected every day into the sample for a week. After that, the sample was kept at the same temperature and pressure for three weeks. This marked the end of the dynamic ageing process. Subsequently, the sample was aged statically by placing it inside a sealed crude oil bath for four months at 80 ^o^C.

Table S2

Crude oil properties. Data from [Alhammadi, et al. ^1^](#_ENREF_1).

|  | Crude oil | Units |
| --- | --- | --- |
| Density at 21 ^o^C | 830 ± 5 | kg/m^3^ |
| Saturates | 55.25 | wt% |
| Aromatics | 38.07 | wt% |
| Resins | 6.22 | wt% |
| Asphaltenes | 0.46 | wt% |
| Total Acid Number | 0.24 | mg KOH/g |
| Total Base Number | 356 | ppm |

**Flow apparatus and experimental procedure**

The flow loop used to perform the experiment is shown in Fig. S1. The apparatus consisted of four Teledyne Isco pumps, a Hassler type carbon fibre coreholder, a micro-CT enclosure and a Parr stirred reactor. The reactor was used to equilibrate the rock, brine and scCO_2_ at the experimental conditions, 70 ^o^C and 10.85 MPa, for 24 h prior to conducting the experiment. The rock used in the reactor is from the same formation as the sample. This step is essential to avoid the dissolution of the rock by the formation of acidic brine, a product of scCO_2_ and brine mixing during the experiment. All the injected volumes of scCO_2_ and brine in the rock were equilibrated.

The experimental procedure was divided into two parts: (i) sample preparation and (ii) flooding sequence. The sample preparation step involved removing the sample from the crude oil bath, wrapping it with aluminium foil and placing it inside a Viton sleeve. The top and bottom of the rock sample were then connected to steel end fitting that were connected to the flow lines, see Fig. S1. This configuration was assembled inside the coreholder and the coreholder was then placed inside the micro-CT enclosure. The reactor, pumps, flow lines and valves were then connected to the coreholder as shown in Fig. S1. The sample was first flushed with 50 pore volumes of doped decane (80% wt decane + 20% wt iododecane mixture) at a rate of 0.1 mL/min to displace all the crude oil in the sample. During this step, the temperature was set to 70 ^o^C and the pressure was raised gradually to the experimental pressure, 10.85 MPa. A flexible heating jacket was wrapped around the coreholder to elevate the temperature and a thermocouple line that was connected to a PID controller was placed next to the sample to control the temperature during the experiment. A confining pressure of 12.85 MPa was applied to ensure the flow is restricted to the vertical direction and prevent any fluid bypassing the along the sample walls.

The flooding sequence in the experiment follows a water-alternating-gas (WAG) injection strategy to mimic the typical displacement sequence encountered in oilfields[^6^](#_ENREF_6). The fluids were injected from the bottom of the rock in this order: (i) first water flooding [WF1]; (ii) gas injection [GI], (iii) second water flooding [WF2]. All injections were performed under capillary dominated conditions to mimic subsurface flow conditions, as opposed to the displacement being controlled by viscous forces. At each injection, one pore volume of fluid was injected into the rock at a rate of 0.005 mL/min, see Table S3. After the experiment, the rock was cleaned and dried at 60 ºC in vacuum for three days to prepare the sample for acquisition of a dry scan.


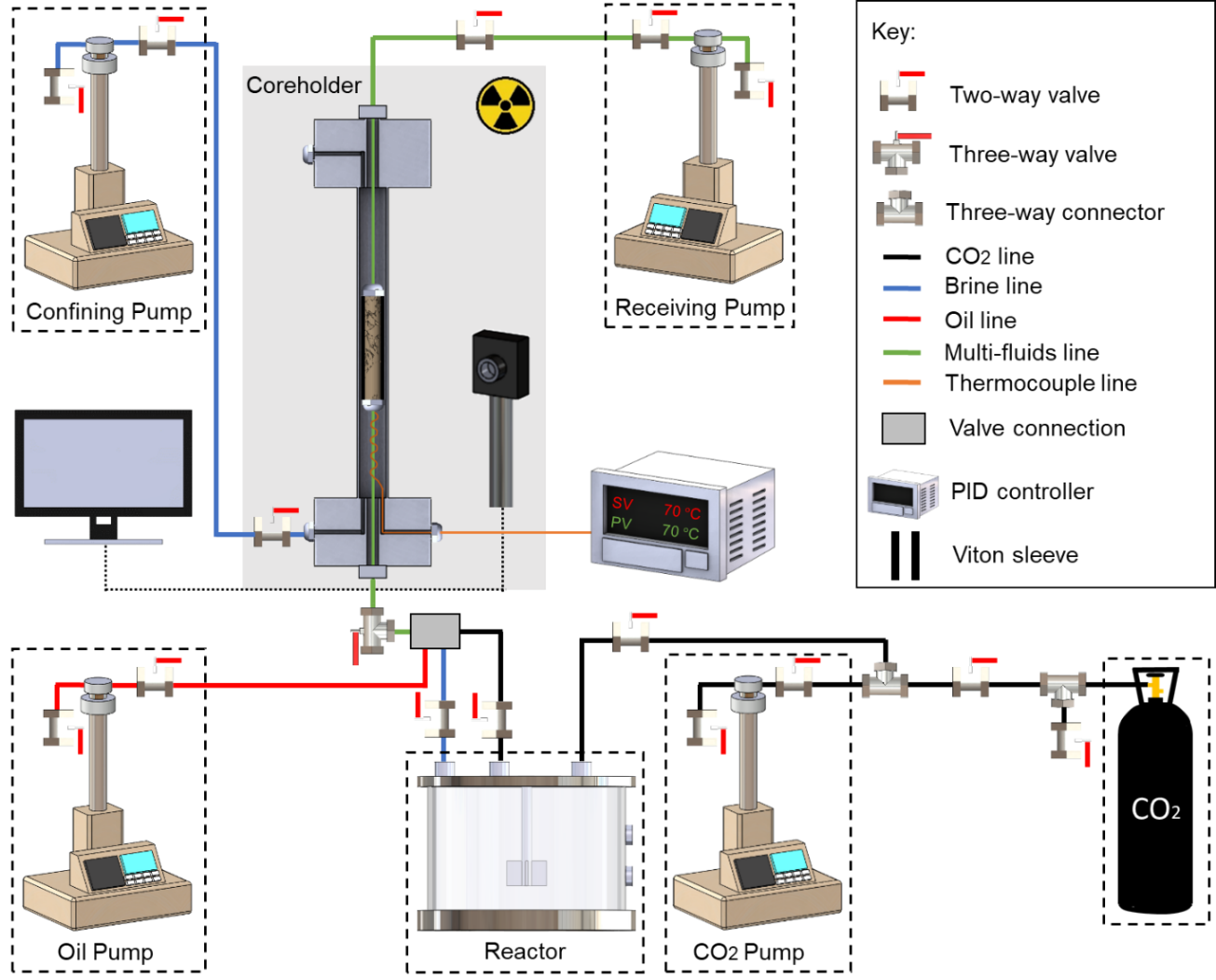


Figure S1 | The high temperature, high pressure flow apparatus used to perform the experiment. The shaded area represents the micro-CT scanner. Experimental conditions are 10.85 MPa and 70 ºC. The flow apparatus consisted of four syringe pumps, stirred reactor, CO_2_ cylinder, coreholder. The 3D models were created using SOLIDWORKS 2019 (<https://www.solidworks.com/>).

Table S3

Details of the experimental injection sequence. PV stands for the pore volume of the sample. The capillary number was calculated using *Ca* = $\mu$*q*/$\sigma$, where $\sigma$ is the interfacial tension between oil and water for water injection, and gas and oil for gas injection, $\mu$ is the viscosity of the displacing (injected) fluid and *q* is the Darcy velocity. $\sigma$ and $\mu$are shown in Table S1, while *q* is calculated by dividing the flow rate by the cross-sectional area of the sample (109 mm^2^).

| Injection sequence | PV injected | Flow rate (mL/min) | Capillary number |
| --- | --- | --- | --- |
| First water flooding [WF1] | 1 | 0.005 | 1.38$\times$10^-7^ |
| Gas injection [GI] | 1 | 0.005 | 1.18$\times$10^-7^ |
| Second water flooding [WF2] | 1 | 0.005 | 1.38$\times$10^-7^ |

**Image acquisition**

The ZEISS Xradia 510 Versa micro-CT scanner was used to acquire high-resolution three-dimensional images of the reservoir rock and the fluids within after each injection. The photon energy range was set to 20-80 keV in the micro-CT with a power of 7 W. The system was allowed to reach equilibrium for two hours before acquiring the scans.

Scans with two resolutions were acquired after each injection: first, a lower resolution scan of 3.57 µm per voxel of the whole sample (1652×1652×6974 voxels) was acquired. Then, a scan of higher resolution, 1.82 µm voxel size, was acquired at the centre of the sample only (1483×1483×1758 voxels), see Fig. S2. The 3.57 µm scans were used to characterize fluid saturations and pore occupancy, while the 1.82 µm scans were used to determine connectivity; the flow simulations were performed on a subset of these images. Details regarding the imaging parameters used in the micro-CT are provided in Table S4.


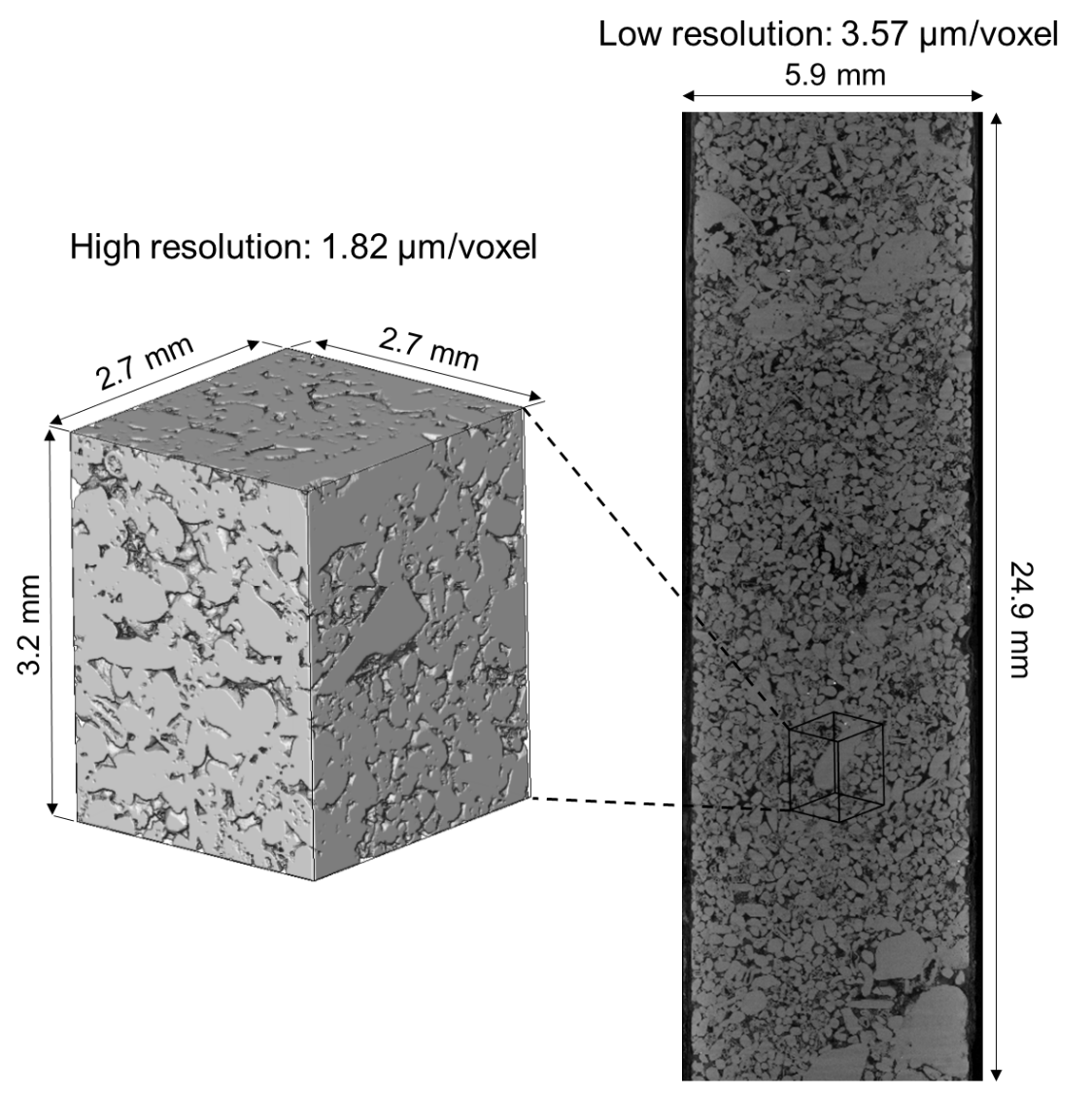


Figure S2 | A schematic showing the location of the high-resolution scan (1483×1483×1758 voxels), 1.82 µm voxel size, (left) relative to the lower-resolution scan (1652×1652×6974 voxels), 3.57 µm voxel size, (right) of the whole sample. The images shown are of the dry scan of the sample.

Table S4

Micro-CT imaging parameters for the 3.57 µm scan (LR) and the 1.82 µm scan (HR) after each injection step. IC stands for initial reservoir conditions.

| Scan | Projections (LR-HR) | Exposure time [s] (LR-HR) | Scanning time [min] (LR-HR) |
| --- | --- | --- | --- |
| Dry | 3201 - 5001 | 1.54 – 4 | 146 × 4 – 540 × 1 |
| IC | 3201 - 5001 | 1.54 – 4 | 146 × 4 – 540 × 1 |
| WF1 | 3201 - 5001 | 2.00 – 4 | 162 × 4 – 540 × 1 |
| GI | 3201 - 5001 | 2.00 – 4 | 162 × 4 – 540 × 1 |
| WF2 | 3201 - 5001 | 2.00 – 4 | 162 × 4 – 540 × 1 |

**Image segmentation**

The lower resolution images of the whole sample, 3.57 µm voxel size, were segmented using the seeded watershed algorithm[^7^](#_ENREF_7). The seeded watershed algorithm locates the seeds of each fluid phase first, i.e. locations of the pore space with high certainty of the present phase. These seeds are then grown proportionally until they reach a boundary, where two fluid phases meet. At that point, the algorithm detects the intensity gradient of each phase in the pore-scale image and assigns the voxels accordingly to avoid misclassification of the fluid phases at the boundaries. This overcomes errors induced by the partial volume effect during the segmentation process[^8^](#_ENREF_8). A non-local means filter was applied to the 3.57 µm voxel size images prior to segmenting them to smooth the grey scale images and improve the watershed segmentation[^9^](#_ENREF_9). Watershed segmentation provides a rapid and accurate segmentation of the micro-CT images; therefore, it was applied to the large 3.57 µm images of the whole rock.

The higher resolution images, 1.82 µm voxel size, were segmented using machine learning-based trainable WEKA segmentation method[^10^](#_ENREF_10). No filter was applied prior to segmenting the images to avoid averaging of voxel values especially at the fluid-fluid contacts to preserve the features of each phase. WEKA segmentation was chosen to segment the high-resolution images as it preserves the shape of the interface between the phases, facilitating more accurate characterization of flow properties and thicknesses of gas layers[^11^](#_ENREF_11)^,^[^12^](#_ENREF_12). The classifier was trained by manually selecting voxels that belong to the oil, rock, brine and gas phases from the micro-CT images. During classifier training, the fast-random algorithm was used alongside the mean and variance texture filters. The trained classifier was then applied to the raw micro-CT images to segment the oil, gas, rock and water phases. Fig. S3 shows raw and segmented images of the higher resolution scans, 1.82 µm. WEKA is very CPU intensive, hence, it was not possible to apply it to the large 3.57 µm images of the whole sample.


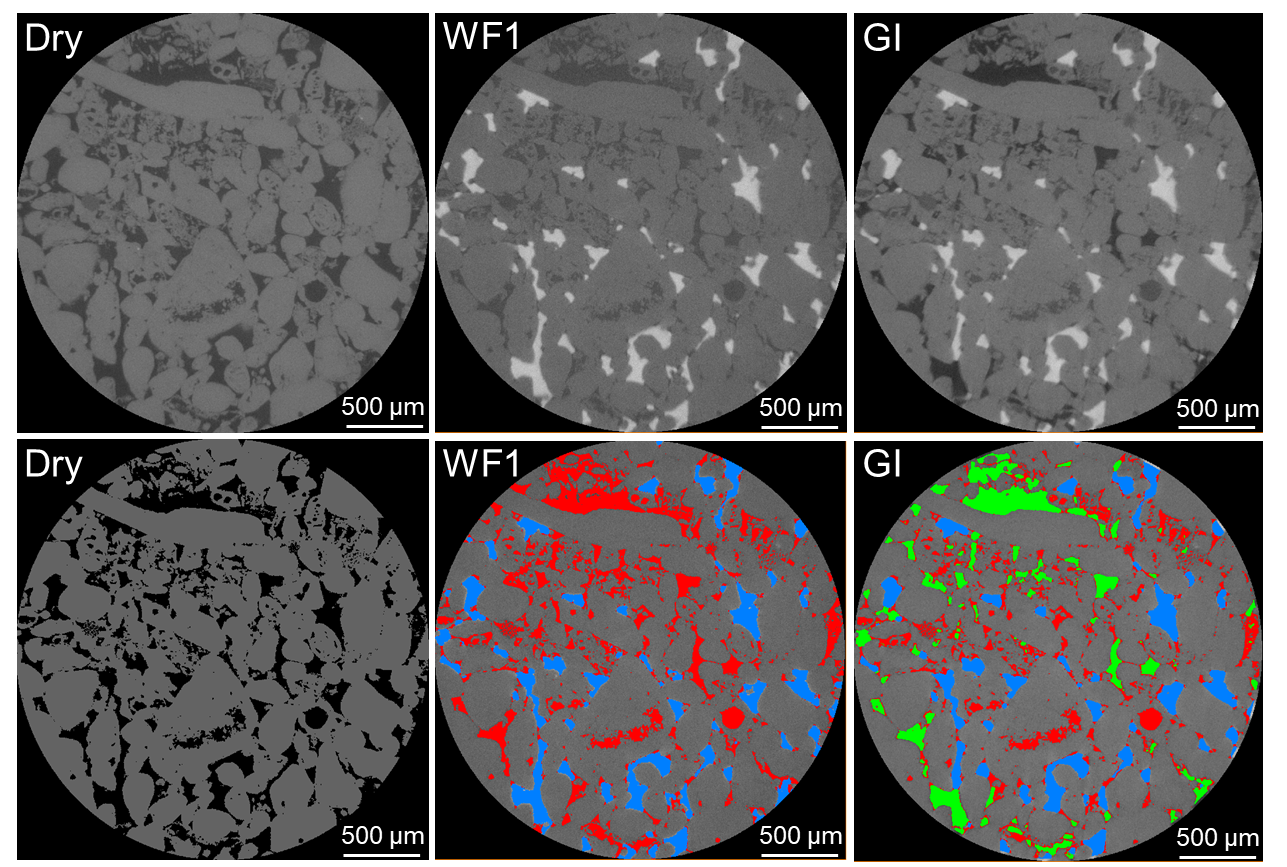


Figure S3 | Images of the higher resolution scans, 1.82 µm voxel size, showing (top row, from left to right) a raw dry scan of the rock, a raw image after the first waterflooding (WF1) and a raw image after gas injection (GI). (bottom row) shows the segmentation of the images in the top row, segmented using WEKA segmentation method. These images were selected to show the accuracy of segmentation for two, three and four phases. In the raw images, gas is shown in black, rock in dark grey, oil in light grey and water in white. In the segmented images, gas is shown in green, rock in grey, oil in red and water in blue.

**Relative permeability calculations**

Each phase was segmented independently, and the Navier-Stokes equations were solved for the flow field of each phase. We used the high-resolution images for the relative permeability calculations. We assumed no flow boundary conditions at the solid surface and the interfaces with other phases, and applied a constant pressure drop across the largest connected cluster in the images, see Fig. S4. We used OpenFoam, a finite element method, to solve for the flow, see[^13^](#_ENREF_13) for more details. Then, using Darcy’s law the permeability of the phase was computed from the ratio of the total flow per unit area times the viscosity to the pressure gradient. This was compared to the absolute permeability, computed when all the pore space was assumed to be filled with one phase. The relative permeability is the ratio of the phase permeability to the absolute permeability.

Since the largest connected cluster of the gas phase after gas injection did not span the system, we cropped all the images to have the same size as the connected gas cluster in GI, indicated by the horizontal lines in Fig. S4, and computed flow on these cropped images of size 1483×1483×1000 voxels. This will tend to over-estimate the relative permeability for the gas phase after GI, since the phase may indeed be disconnected (relative permeability of zero), or only connected through regions that cannot be resolved in the scan.


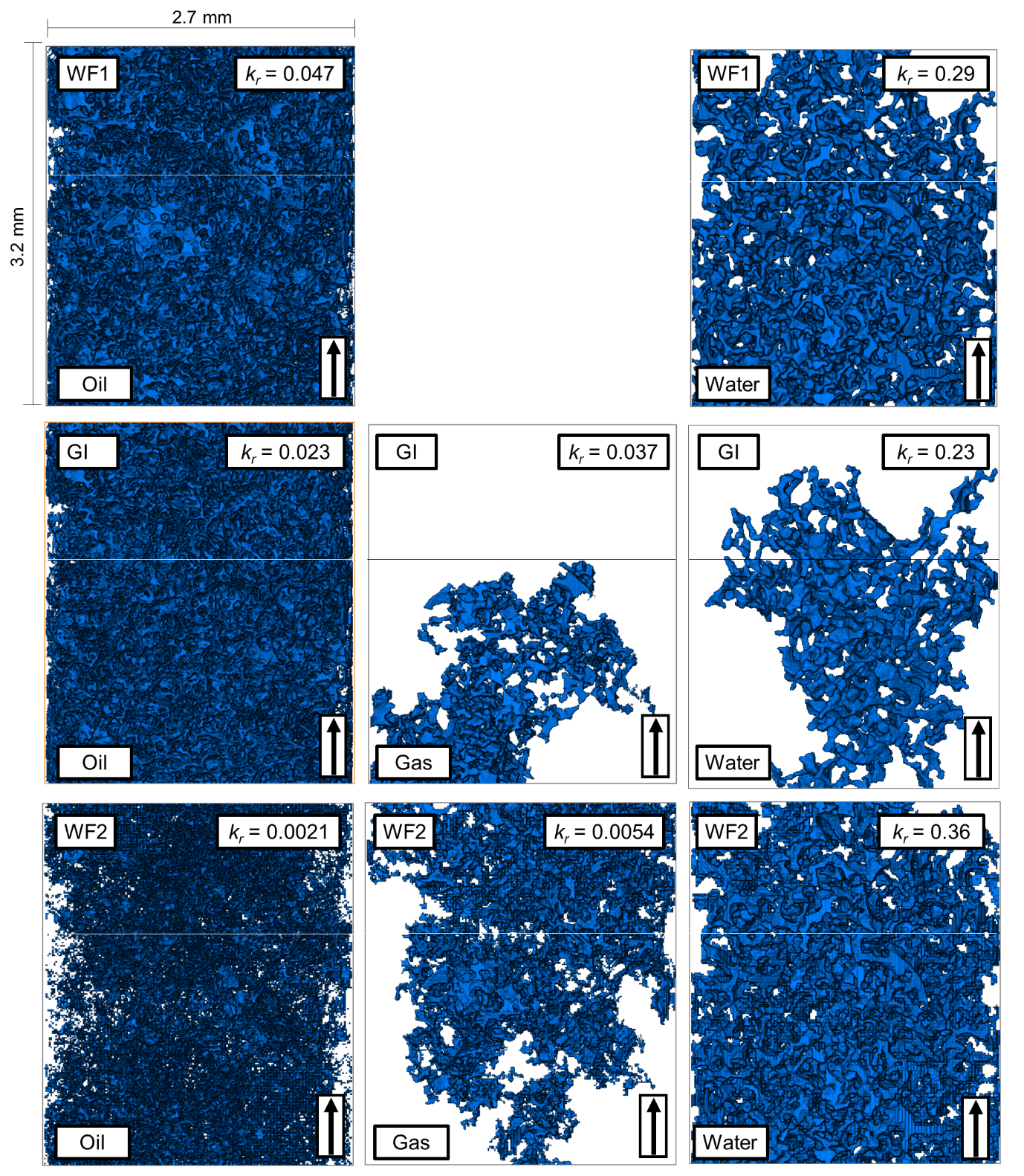


Figure S4 | A series of 2D images showing the connected clusters of oil, gas and water phases for which the relative permeability was measured during: (first row) first waterflooding (WF1), (second row) gas injection (GI) and (iii) second waterflooding (WF2). The relative permeabilities were quantified on images of resolution of 1.82 µm/voxel and size of 1483×1483×1000 voxels: the region beneath the horizontal line was used to quantify the relative permeability. The clusters were plotted using Avizo 9.5 software (<https://www.fei.com/software/amira-avizo/>).

References

1 Alhammadi, A. M., AlRatrout, A., Singh, K., Bijeljic, B. & Blunt, M. J. In situ characterization of mixed-wettability in a reservoir rock at subsurface conditions. *Scientific Reports* **7**, 10753, doi:10.1038/s41598-017-10992-w (2017).

2 Alhosani, A. *et al.* In situ pore-scale analysis of oil recovery during three-phase near-miscible CO2 injection in a water-wet carbonate rock. *Advances in Water Resources* **134**, 103432, doi:<https://doi.org/10.1016/j.advwatres.2019.103432> (2019).

3 Georgiadis, A. *et al.* Interfacial tension measurements and modelling of (carbon dioxide+n-alkane) and (carbon dioxide+water) binary mixtures at elevated pressures and temperatures. *The Journal of Supercritical Fluids* **55**, 743-754, doi:<https://doi.org/10.1016/j.supflu.2010.09.028> (2010).

4 Heidaryan, E., Hatami, T., Rahimi, M. & Moghadasi, J. Viscosity of pure carbon dioxide at supercritical region: Measurement and correlation approach. *The Journal of Supercritical Fluids* **56**, 144-151, doi:<https://doi.org/10.1016/j.supflu.2010.12.006> (2011).

5 NIST. *Reference Fluid Thermodynamic and Transport Properties Database (REFPROP)*, <<https://www.nist.gov/srd/refprop>> (2019).

6 Lake, L. W. Enhanced oil recovery. Elsevier (1989).

7 Jones, T. R., Carpenter, A. & Golland, P. in *Computer Vision for Biomedical Image Applications.* (eds Yanxi Liu, Tianzi Jiang, & Changshui Zhang) 535-543 (Springer Berlin Heidelberg).

8 Brown, K., SchlÜTer, S., Sheppard, A. & Wildenschild, D. On the challenges of measuring interfacial characteristics of three-phase fluid flow with x-ray microtomography. *Journal of Microscopy* **253**, 171-182, doi:10.1111/jmi.12106 (2014).

9 Buades, A., Coll, B. & Morel, J.-M. Nonlocal Image and Movie Denoising. *International Journal of Computer Vision* **76**, 123-139, doi:10.1007/s11263-007-0052-1 (2008).

10 Arganda-Carreras, I. *et al.* Trainable Weka Segmentation: a machine learning tool for microscopy pixel classification. *Bioinformatics* **33**, 2424-2426, doi:10.1093/bioinformatics/btx180 (2017).

11 Kaynig, V., Fuchs, T. & Buhmann, J. M. in *2010 IEEE Computer Society Conference on Computer Vision and Pattern Recognition.* 2902-2909.

12 Rudyanto, R. D. *et al.* Comparing algorithms for automated vessel segmentation in computed tomography scans of the lung: the VESSEL12 study. *Medical Image Analysis* **18**, 1217-1232, doi:<https://doi.org/10.1016/j.media.2014.07.003> (2014).

13 Raeini, A. Q., Blunt, M. J. & Bijeljic, B. Modelling two-phase flow in porous media at the pore scale using the volume-of-fluid method. *Journal of Computational Physics* **231**, 5653-5668, doi:<https://doi.org/10.1016/j.jcp.2012.04.011> (2012).
